# Type I-conventional dendritic cells support the progression of multiple myeloma in the bone marrow

**DOI:** 10.3389/fimmu.2024.1444821

**Published:** 2024-10-15

**Authors:** Sayaka Suzuki, Kazuma Komiya, Shogo Tsuda, Miya Yoshino, Tsuneyasu Kaisho, P. Leif Bergsagel, Koji Kawamura, Tetsuya Fukuda, Koji Tokoyoda

**Affiliations:** ^1^ Division of Immunology, School of Life Science, Faculty of Medicine, Tottori University, Yonago, Japan; ^2^ Division of Hematology and Clinical Laboratory Medicine, Department of Multidisciplinary Internal Medicine, School of Medicine, Faculty of Medicine, Tottori University, Yonago, Japan; ^3^ Department of Immunology, Institute of Advanced Medicine, Wakayama Medical University, Wakayama, Japan; ^4^ Division of Hematology/Oncology, Mayo Clinic in Arizona, Scottsdale, AZ, United States; ^5^ Division of Hematology, Department of Medicine, Showa University Fujigaoka Hospital, Yokohama, Japan

**Keywords:** conventional dendritic cell, multiple myeloma, tumor immune microenvironment, CD103, bone marrow

## Abstract

**Purpose:**

Type I conventional dendritic cells (cDC1s) play a key role in priming anti-tumor cytotoxic T cells and inducing immune tolerance for self-antigens and tumor antigens. However, it remains unclear whether cDC1 has a protective or pathogenic role in multiple myeloma. We investigated a role of cDC1 in myeloma progression.

**Methods:**

A myeloma mouse model was performed by intravenous transplantation of Vk*MYC myeloma cells into XCR1-Diphtheria toxin receptor (DTR) knock-in or wild-type mice. Following injection with Diphtheria toxin (DT), monoclonal (M)-proteins and myeloma cells were analyzed by ELISA and flow cytometry.

**Results:**

Here we show that inducible depletion of cDC1 after myeloma transplantation markedly suppressed the progression of myeloma in the bone marrow and extramedullary sites, such as the spleen. cDC1 appeared in the bone marrow and spleen of myeloma-transplanted mice, which highly expressed CD103 and lowly produced interleukin (IL)-12. Consequently, the frequencies of exhausted CD8 T cells and regulatory T cells significantly decreased in the bone marrow of cDC1-depleted mice.

**Conclusions:**

cDC1 supports the progression of myeloma inducing exhausted CD8 T cells and regulatory T cells.

## 1 Introduction

Multiple myeloma is a plasma cell malignancy, that causes multiple immunodeficiencies in cancer immunity and infections. Repeated relapses lead to a poor prognosis not only by disease progression but also by opportunistic infections due to immunodeficiency. Although novel therapies have improved survival rates, it is still impossible to completely cure the disease. Some immunotherapies, such as chimeric antigen receptor T-cell therapies and bispecific antibodies, have shown positive effects in practical use for the treatment of highly active relapsed refractory multiple myeloma, suggesting that a therapeutic strategy utilizing the immune system is effective. Tumor-specific CD8 T cells play an important role in cancer immunity; however, they have been observed to be in an exhausted state in many types of malignancy. cDC1s can incorporate tumor-derived proteins and prime naïve CD8 T cells via cross-presentation as components of the tumor immune microenvironment (TiME). Recent studies have shown that dysfunction of cDC1 develops in solid malignancies (1–3). Multiple myeloma beneficially alters the TiME for survival and progression, such as by increasing the number of regulatory T cells, exhausted CD8 T cells, and myeloid-derived suppressor cells, and decreasing that of cytotoxic CD8 T cells (4–6). However, whether cDC1 has a positive or negative role in multiple myeloma remains unclear. This study aimed to investigate whether cDC1 contributes protectively or pathogenically to myeloma progression in a Vk*MYC myeloma mouse model. We found that cDC1 supports myeloma progression by increasing the number of exhausted CD8 T cells and regulatory T cells.

## 2 Materials and methods

### 2.1 Mice and cell line

C57BL/6J (Charles River) and XCR1-DTR mice (7) were maintained under specific pathogen-free conditions at the animal facility of the Advanced Medicine and Translational Research Center of Tottori University. All experiments were approved by the Animal Experiment Committee of Tottori University. In a mouse myeloma model, Vk*MYC myeloma cells [Vk12653 cells (8)] were intravenously transplanted into male mice aged 6-8 weeks at a density of 1.0×10^7^ cells per mouse. To deplete cDC1, XCR1-DTR mice were intraperitoneally injected six times with DT (BioAcademia) at a dose of 40 ng/g body weight for the first dose 10-12 days after transplantation (0.2-0.8 mg/mL M-protein in serum), with follow-up doses of 4 ng/g every other day.

### 2.2 Enzyme-linked immunosorbent assay (ELISA)

Vk*MYC cells secrete M-protein, a subclass of IgG2b-κ. To measure serum IgG2b titers, serum samples were incubated in ELISA plates (Corning) coated with goat anti-mouse Ig, F(ab’)_2_ fragment (115-005-072, Jackson Immuno Research). The IgG2b isotype was determined using alkaline phosphatase-conjugated antibodies to mouse IgG2b and pNPP substrate (Sigma-Aldrich) and was measured using the Sunrise Rainbow (TECAN). Purified mouse IgG2b-κ (BioLegend) was used to plot the standard curve.

### 2.3 Flow cytometry

Single-cell suspensions were prepared from the spleen and femurs of individual mice after digestion with 1 mg/mL collagenase D (Roche) and 50 U/mL deoxyribonuclease I (Tokyo Chemical Industry) at 37°C. Cell viability was assessed using trypan blue exclusion. The cells were stained with antibodies against CD11c, XCR1, CD4, CD83, CD86, CD103, CXCR4, T-cell immunoglobulin mucin domein-3 (Tim3), programmed cell death-1 (PD-1), and programmed cell death-1 ligand 1 (PD-L1) (BioLegend), CD11b, CD8a, and CD155 (BD Biosciences), B220 (Invitrogen or Miltenyi Biotec), B- and T-lymphocyte attenuator (BTLA) and CCR7 (Miltenyi Biotec), CD80 (Invitrogen), and major histocompatibility complex (MHC) class II and integrin αV (in-house). The dead cells were stained with propidium iodide (AAT Bioquest). For intracellular staining, cells were stimulated with 1 μg/mL lipopolysaccharide (LPS) (L4391, Sigma-Aldrich) with 4 μg/mL Brefeldin A (Sigma-Aldrich) for 4 hours and stained with anti-IL-12 antibody (BD Biosciences) after treatment with BD Cytofix/Cytoperm buffer (BD Biosciences) according to the manufacturer’s protocol. To detect regulatory T cells, intranuclear staining was performed using a Foxp3 Transcription Factor staining buffer kit (Invitrogen) and anti-FOXP3 antibody (BioLegend). All cytometric data were collected using a flow cytometer (FACS Celesta; BD Biosciences) and analyzed using FlowJo software (BD Biosciences).

### 2.4 Statistical analysis

Unpaired *t*-test was used to compare groups with equal variance. For serum IgG2b growth curves, significance was determined using a two-way analysis of variance. Statistical analyses were performed using GraphPad Prism software (GraphPad Software Inc.). The level of significance is indicated as *p<0.05, **p<0.01, and ***p<0.001, and is described in the figure legends.

## 3 Results

### 3.1 Depletion of cDC1 suppresses myeloma progression

**Figure 1:**
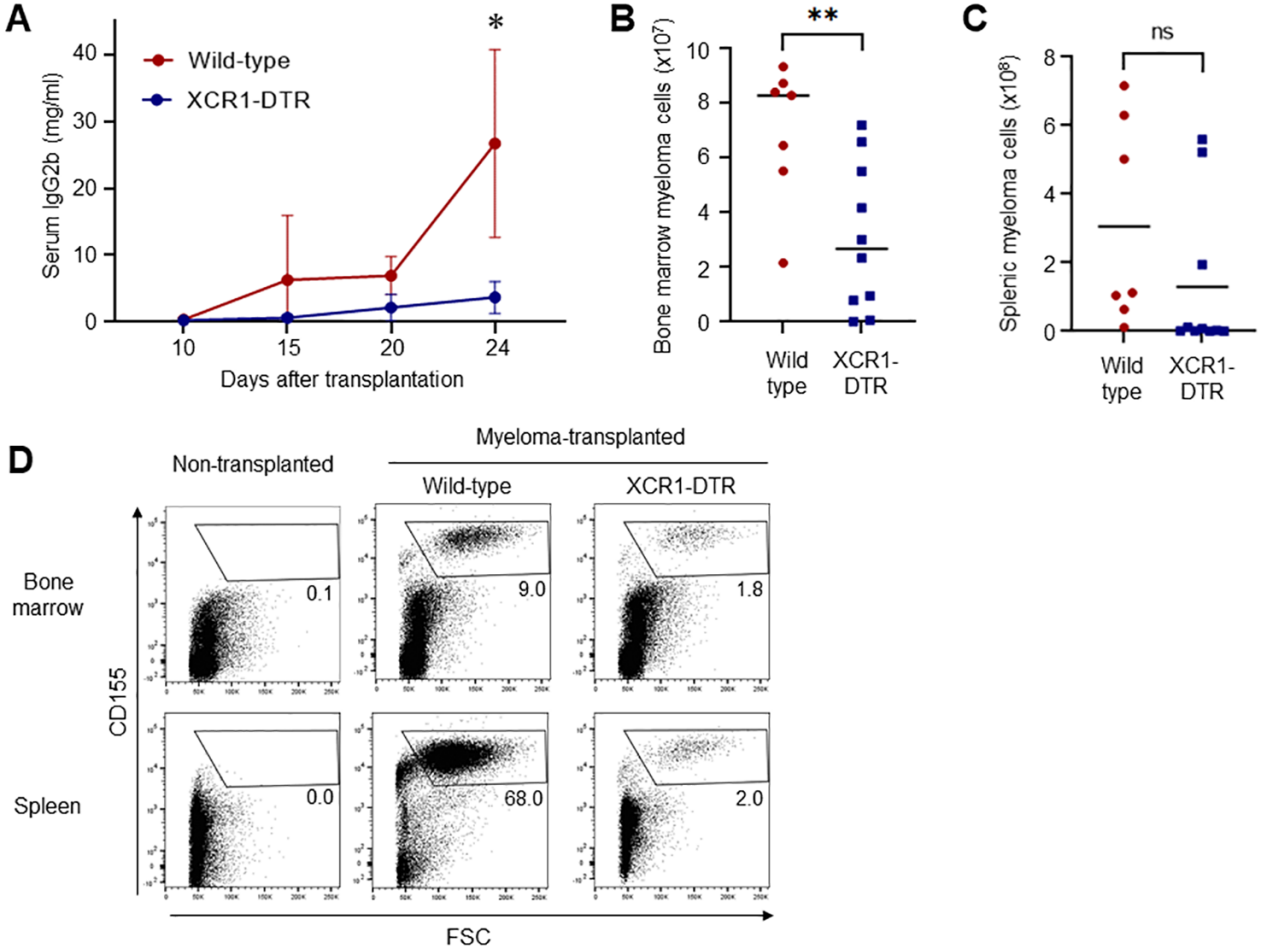
Depletion of cDC1 suppresses myeloma progression. **(A)** Loss of cDC1 decreases the serum titers of M-protein (IgG2b). XCR1-DTR and wild-type mice were transplanted with Vk*MYC cells and injected with DT 12, 14, 16, 18, 20 and 22 days after transplantation. Sera on days 10, 15, 20 and 24 were analyzed via ELISA. Data were merged from four independent experiments. Data are shown as mean ± SD. N=10-16. ns: not significant, *:p<0.05. **(B-D)** Loss of cDC1 decreases the numbers of myeloma cells in the bone marrow and spleen. Following transplantation and DT administration as well as **(A)**, CD155^high^ myeloma cells in the bone marrow **(B)** and spleen **(C)** were counted via flow cytometry 24 days after transplantation. Numbers **(B, C)** and dot plots in live cells **(D)** are shown. Data were merged from four independent experiments. N=7-10. ns, not significant, **:p<0.01.

To test whether cDC1 supports or inhibits the progression of myeloma, we used a Vk*MYC myeloma mouse model that can be used to monitor the tumor based on CD155 and IgG2b expression, and the XCR1-DTR mouse line that can inductively deplete cDC1 in the body. Because the chemokine receptor XCR1 is specifically expressed by cDC1, injections of DT selectively deplete cDC1 in the body. More than 95% of cDC1s is depleted in the spleen (Ref. 7, data not shown). Transplanted Vk*MYC myeloma cells expanded in the bone marrow and secreted IgG2b as M-protein. After expansion, the myeloma cells proliferated exponentially in the spleen (8). XCR1-DTR and wildtype mice were transplanted with Vk*MYC cells and injected with DT every other day, starting 12 days after transplantation. Interestingly, depletion of cDC1 drastically suppressed the production of M-protein (**Figure 1A**) and the expansion of myeloma cells for CD155^high^ large cells in the bone marrow and spleen, as revealed by flow cytometry (**Figures 1B–D**). In addition, DT injection from Day 0 failed to reduce the numbers of bone marrow and splenic myeloma cells (**Supplementary Figure 1**).

### 3.2 Appearance of mature cDC1 in the bone marrow of myeloma-transplanted mice

**Figure 2:**
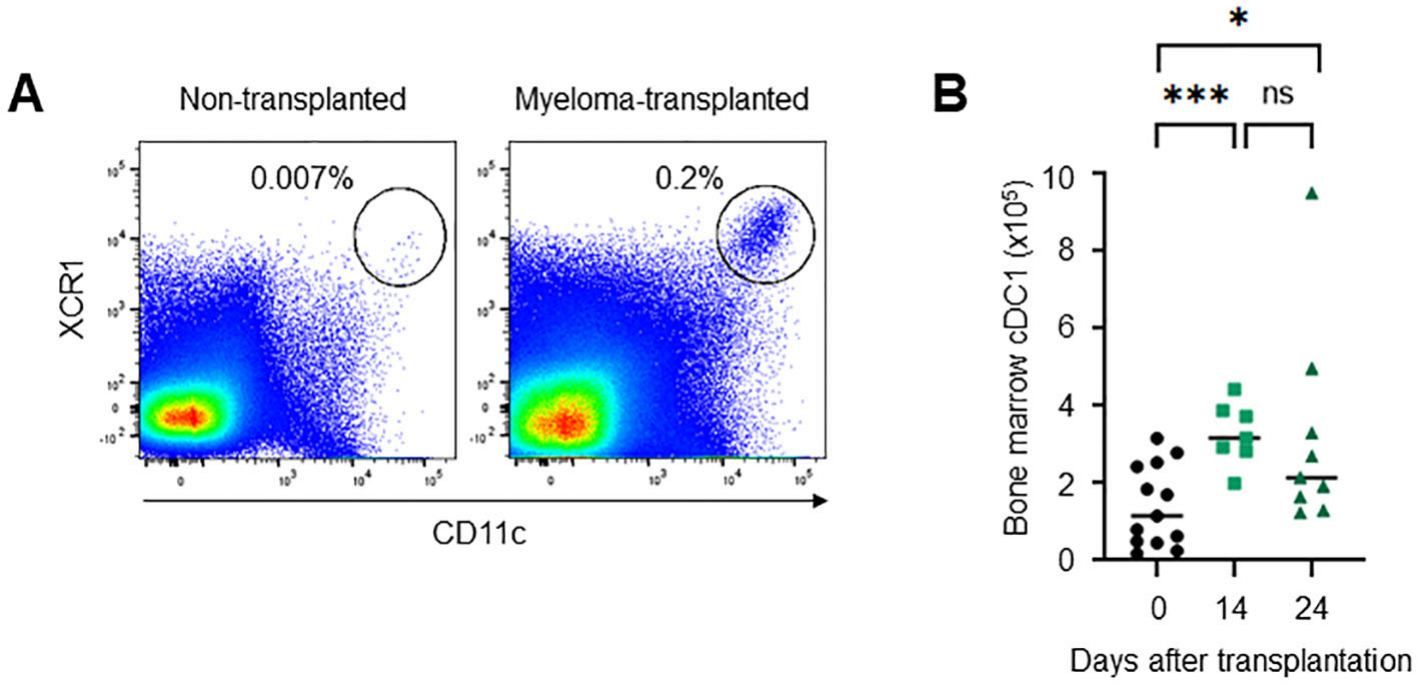
Mature cDC1s are present in the bone marrow of myeloma-transplanted mice. The number of XCR1^+^CD11c^high^ cells increased in the bone marrow of myeloma-transplanted mice. Dot plots on days 0 and 24 **(A)** and the numbers on days 0, 14, and 24 **(B)** of bone marrow XCR1^+^CD11c^high^ cells analyzed via flow cytometry. Data were merged from four independent experiments. N=7-13. ns, not significant; *:p<0.05, ***:p<0.001.

**Figure 3:**
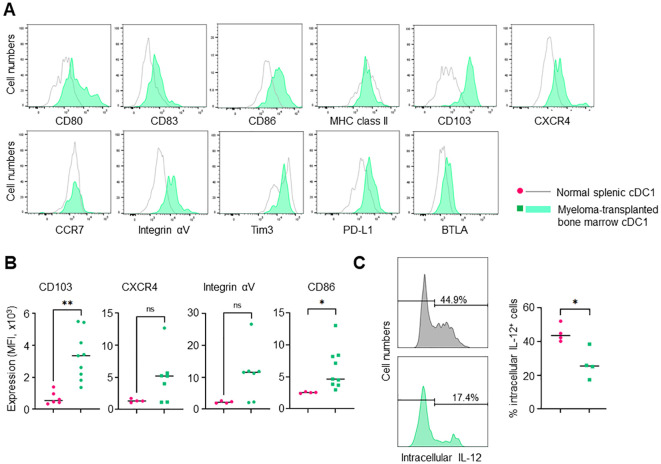
Phenotypes of myeloma-induced bone marrow cDC1. **(A)** The expression of DC maturation makers in myeloma-induced cDC1. The histograms show the expression of CD80, CD83, CD86, MHC class II, CD103, CXCR4, CCR7, integrin αV, Tim3, PD-L1 and BTLA in bone marrow cDC1s (XCR1^+^CD8a^+^CD11c^high^) of myeloma-transplanted mice (green, filled) and splenic cDC1s of normal mice (grey, empty) 24 days after transplantation. **(B)** CD103 expression is significantly higher in myeloma-induced cDC1 than in normal cDC1. The graph shows the mean fluorescence intensity (MFI) of CD103, CXCR4, integrin αV and CD86 in bone marrow cDC1s of myeloma-transplanted mice (green) and splenic cDC1s of normal mice (red). **(C)** Myeloma-induced cDC1 suppressed IL-12 production. The histogram shows the expression of intracellular IL-12 in bone marrow XCR1^+^CD11c^high^ cDC1s of myeloma-transplanted mice (green, filled) and splenic cDC1s of normal mice (grey, filled) following LPS stimulation with Brefeldin A for 4 hours. The graph shows the proportion of intracellular IL-12 positive cDC1s in bone marrow cDC1s of myeloma-transplanted mice (green) and splenic cDC1s of normal mice (red). Data were merged from three independent experiments. N=4-9. ns, not significant; *:p<0.05, **:p<0.01.

To clarify the role of cDC1 in myeloma progression, we investigated the phenotype of cDC1 in the bone marrow of myeloma- transplanted mice. In general, few mature cDC1s remain in normal bone marrow. Interestingly, mature CD11c^+^CD8a^+^XCR1^+^CD11b^-^ cDC1s accumulated in the bone marrow 14 and 24 days after myeloma transplantation (**Figure 2**). The mature cDC1s expressed some markers for activation (CD80, CD83, CD86 and MHC class II) and migration (CCR7 and CXCR4) as well as splenic cDC1 at steady state (**Figure 3A**). A marker for tissue residency of cDC, CD103 (integrin αE) was expressed eight-fold higher than normal cDC1, which may be considered as a marker of myeloma-restricted cDC1 (**Figure 3B**). Integrin αV, PD-L1, and BTLA are expressed in suppressive and tolerogenic cDC1s inducing regulatory T cells and exhausted T cells (9). Myeloma-induced cDC1 did not significantly expressed molecules that might function as tolerogenic cDC1 (**Figures 3A, B**). To examine whether cDC1 is functional for cellular immunity to myeloma, the expression of the cytokine IL-12, which is involved in Th1 and cytotoxic T cell differentiation, was analyzed using flow cytometry. Myeloma-induced bone marrow cDC1 produced lower IL-12 than normal splenic cDC1 4 hours after stimulation with LPS (**Figure 3C**).

### 3.3 cDC1 alters the proportion of T cell populations in the bone marrow

**Figure 4:**
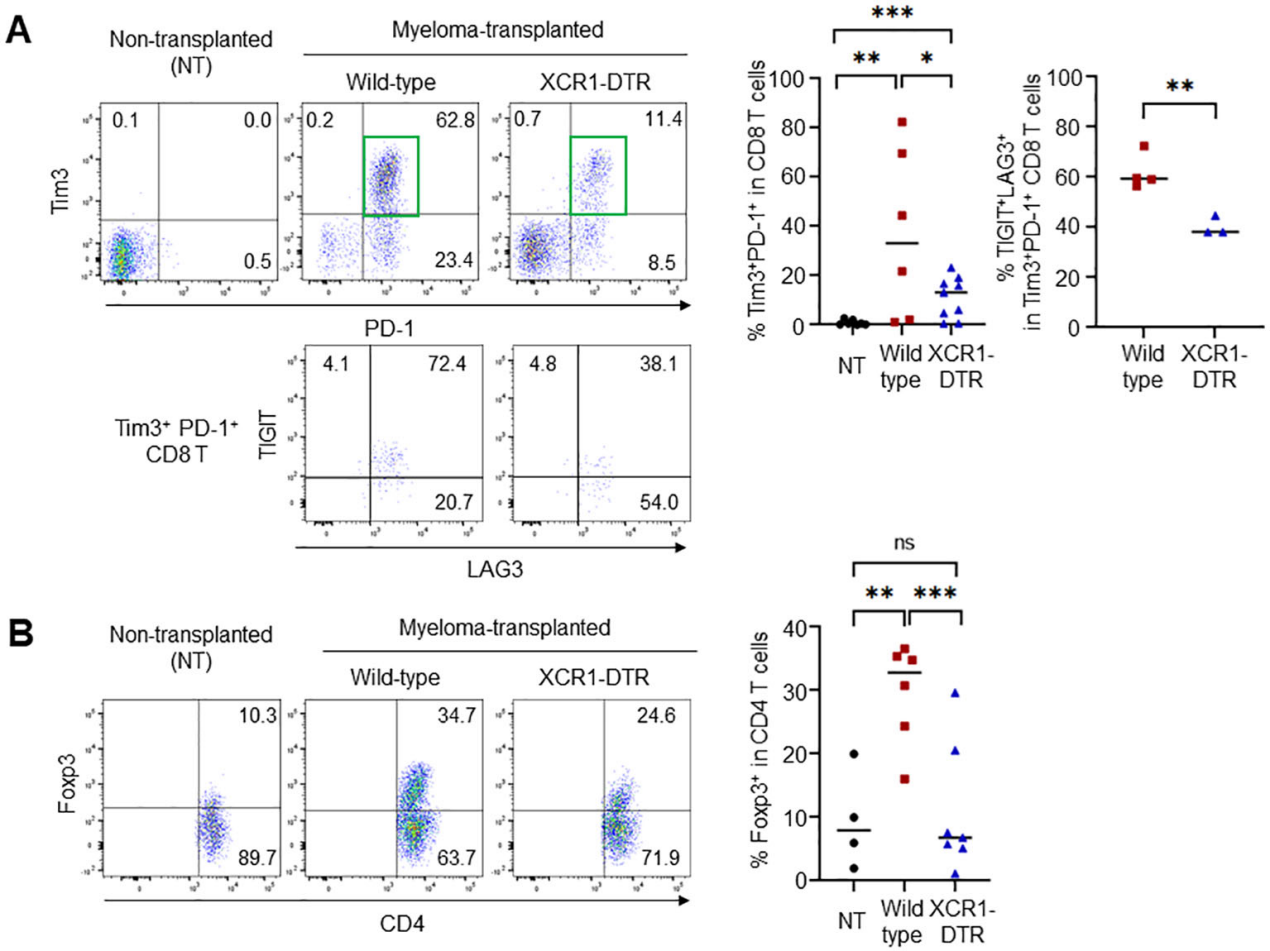
Loss of cDC1 suppresses the generation of exhausted and regulatory T cells. **(A)** cDC1 depletion in myeloma-transplanted mice reduces the frequency of exhausted CD8 T cells. Dot plots (left) and graph (right) show the frequencies of Tim3^+^PD-1^+^ in CD8 T cells and TIGIT^+^LAG3^+^ in Tim3^+^PD-1^+^ CD8 T cells 24 days after transplantation by flow cytometry. **(B)** cDC1 depletion in myeloma-transplanted mice reduces the frequency of regulatory CD4 T cells. Dot plots (left) and graph (right) show the frequencies of Foxp3^+^ in CD4 T cells 24 days after transplantation by flow cytometry. Data were merged from three independent experiments. N=6-9. ns, not significant; *:p<0.05, **:p<0.01, ***: p<0.001.

Tumors increase the number of suppressive T cells, such as exhausted and regulatory T cells. In the Vk*MYC mouse model, frequencies of activated/memory-phenotype CD44^high^CD62L^-^ T cells were increased in myeloma-transplanted bone marrow (**Supplementary Figure 2**). However, suppressive T cells increased 21 days after transplantation (**Figure 4**). Myeloma-induced cDC1s lowly expressed IL-12. To investigate whether cDC1 generates exhausted T cells and regulatory T cells, Tim3^+^PD-1^+^ CD8 T cells and Foxp3^+^ CD4 T cells in the bone marrow were counted after cDC1 depletion. On day 21 after transplantation (11-12 days after DT injection), the increased frequencies of Tim3^+^PD-1^+^ and Foxp3^+^ regulatory T cells by myeloma transplantation were cancelled by the loss of cDC1s (**Figures 4A, B**), whereas those of total CD8, Tim3^-^PD-1^+^ CD8 T, and total CD4 T cells in the bone marrow were comparable. Furthermore, in the Tim3^+^PD-1^+^ CD8 T cells, two inhibitory molecules, TIGIT- and LAG3- double-positive cells were increased by myeloma transplantation and the increment was cancelled by cDC1 depletion (**Figure 4A**). These results suggest that cDC1 is altered by myeloma, induces immunosuppressive T cells, and supports myeloma progression.

### 3.4 cDC1 increases also in the spleen

**Figure 5:**
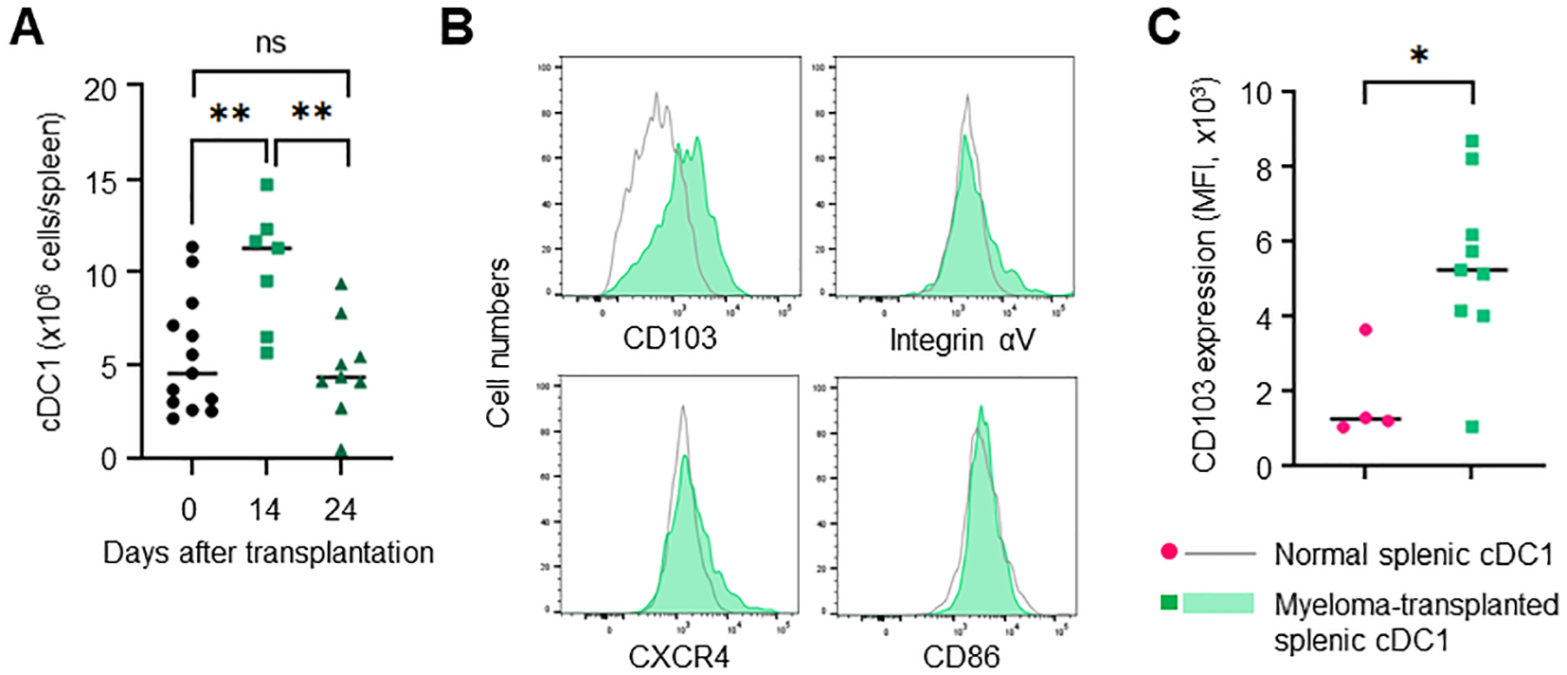
Splenic cDC1 levels are increased in myeloma-transplanted mice. **(A)** cDC1s are transiently increased in the spleen of myeloma-transplanted mice. The numbers of splenic XCR-1+CD11c^high^ cells 0, 14 and 24 days after transplantation were determined via flow cytometry. **(B)** cDC1s of the spleen are distinct from those of the bone marrow in myeloma-transplanted mice. The histograms show the expression of CD103, integrin αV, and CXCR4 in splenic XCR 1+CD11c^high^ cells of myeloma-transplanted mice (blue, filled) and normal mice (grey, empty) 24 days after transplantation by flow cytometry. **(C)** CD103 expression is higher in myeloma-induced cDC1 than in normal cDC1. The graph shows MFI of CD103 in splenic cDC1s of myeloma-transplanted (green) and normal mice (red). Data were merged from two independent experiments. N=4-13. ns: not significant, *: p<0.05, **: p<0.01.

The bone marrow contains few mature cDC1s in a steady state. However, how mature cDC1 accumulates in the bone marrow remains unclear. In the Vk*MYC mouse model, myeloma cells spread in the bone marrow mainly 10-20 days after transplantation and then expanded in the spleen, leading to splenomegaly. In the early phase, for example, 14 days after transplantation, the spleen had few tumors (data not shown). However, the number of cDC1 transiently increased in the spleen (**Figure 5A**). The increased cDC1 highly expressed CD103 as well as bone marrow cDC1 induced by myeloma (**Figures 5B, C**). In the circulation, cDC1 significantly increased on day 24 but not 14 after transplantation (**Supplementary Figure 3**), suggesting that cDC1 is independently increased in the bone marrow and spleen.

## 4 Discussion

### 4.1 cDC1 contributes to myeloma progression

In this study, we showed that mature cDC1 is present in the bone marrow of myeloma-transplanted mice and that depletion of cDC1 suppresses myeloma progression. We also showed that myeloma-induced cDC1 highly express a marker for tissue-residency, CD103, and reduce the production of a cytokine inducing cellular immunity, IL-12. The presence of cDC1 subsequently induces an increase in the numbers of exhausted and regulatory T cells, which contributes to myeloma progression. To the best of our knowledge, this is the first evidence that cDC1 supports the progression of multiple myeloma *in vivo*.

### 4.2 Loss of cDC1 inhibits myeloma progression

Myeloma-induced cDC1 highly expressed CD103 (known as integrin αE). Tumor-infiltering DCs express CD103, are called mature DCs enriched in immunoregulatory molecules, mregDC1 (10, 11). MregDC1 is considered to have a regulatory function, but this remains unclear. CD103 forms dimers with the integrin β7 subunit and binds to E-cadherin expressed mainly on epithelial cells. CD103 is expressed in tissue-resident T cells and functions as a homing/retention molecule in or within peripheral tissues, such as the skin, lungs, and the gut (12). Myeloma cells express E-cadherin and interact with plasmacytoid DC using CD103/E-cadherin for progression (13), suggesting that they directly interact with cDC1 and alter their function.

CD103^+^ cDC1s were also increased in the spleen but not the circulation on day 14 after transplantation, suggesting that splenic and bone marrow cDC1 were independently increased. cDC1s expressed CD103 but not any inhibitory molecules; integrin αV, PD-L1, and BTLA. Myeloma decreased IL-12-expressing functional cDC1 and did not increase suppressive and tolerogenic cDC1 in the bone marrow as an immune-editor. Since cDC1 had no phenotype for tolerogenic cDC1 which induces suppressive T cells directly, functional T cells may be infiltrated by increased cDC1s in the bone marrow and spleen and directly changed into suppressive T cells by myeloma. Myeloma cells were from B cells ad have high affinity to T cells.

### 4.3 cDC1 as a clinical target of multiple myeloma

Our study indicates that loss of cDC1 reverses the immunosuppressive microenvironment and inhibits myeloma progression, suggesting that the presence of cDC1 is harmful in the middle phase of myeloma progression. In clinical trials, vaccination and DC therapies for multiple myeloma have failed to work effectively (14). Our data suggest that DC can be easily altered in the myeloma-induced TiME. Injection of DT into XCR1-DTR mice induced cDC1 depletion in the whole body, not only within the tumor. This means that all cDC1s can be clinically targeted.

cDC1 plays a key role in priming anti-tumor cytotoxicity and inducing immune tolerance. Myeloma cells strongly induce a tolerant microenvironment in the bone marrow, and cDC1 contributes to the immunosuppression of tumors, cooperating with exhausted and regulatory T cells. A frequency of regulatory T cells increases in the bone marrow of Vk*MYC myeloma-transplanted mice and their depletion promotes myeloma progression (15). Taken together, the results suggest that cDC1 edited by myeloma cells expands regulatory T cells and forms an immunosuppressive microenvironment.

## Data Availability

The raw data supporting the conclusions of this article will be made available by the authors, without undue reservation.
