# Supplementary material for: Type I-conventional dendritic cells support the progression of multiple myeloma in the bone marrow
Source: Front Immunol. 2024 Oct 15;15:1444821. doi: 10.3389/fimmu.2024.1444821 (PMC11518711; doi:10.3389/fimmu.2024.1444821)
Supplement: Supplementary file 1 [file DataSheet1.docx]

Supplementary Figures

Suzuki S. et al. Type I-Conventional Dendritic Cells Support the Progression of Multiple Myeloma in the Bone Marrow


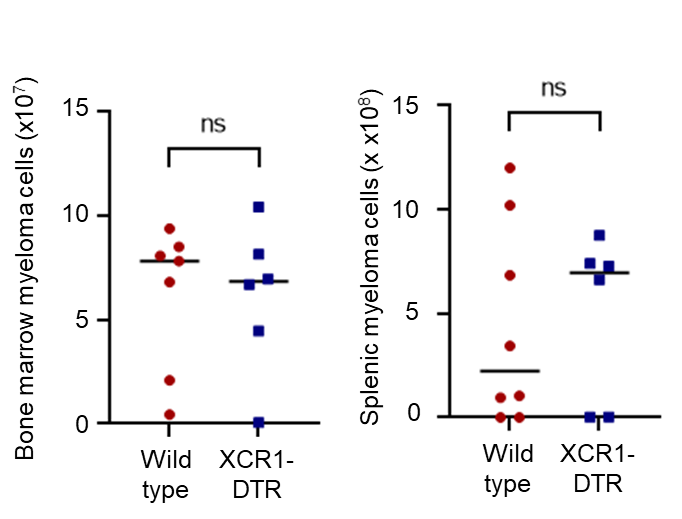


Supplementary Figure 1. Loss of cDC1 in the early phase of myeloma transplantation did not affect myeloma cell expansion.

XCR1-DTR and wild-type mice were transplanted with Vk*MYC cells and injected with DT 0, 2, 4, 6, 8, 10, 12, 14, 16, 18 20 and 22 days after transplantation. CD155^high^ myeloma cells were counted via flow cytometry 24 days after transplantation. Data were merged from two independent experiments. N=6-7. ns: not significant.


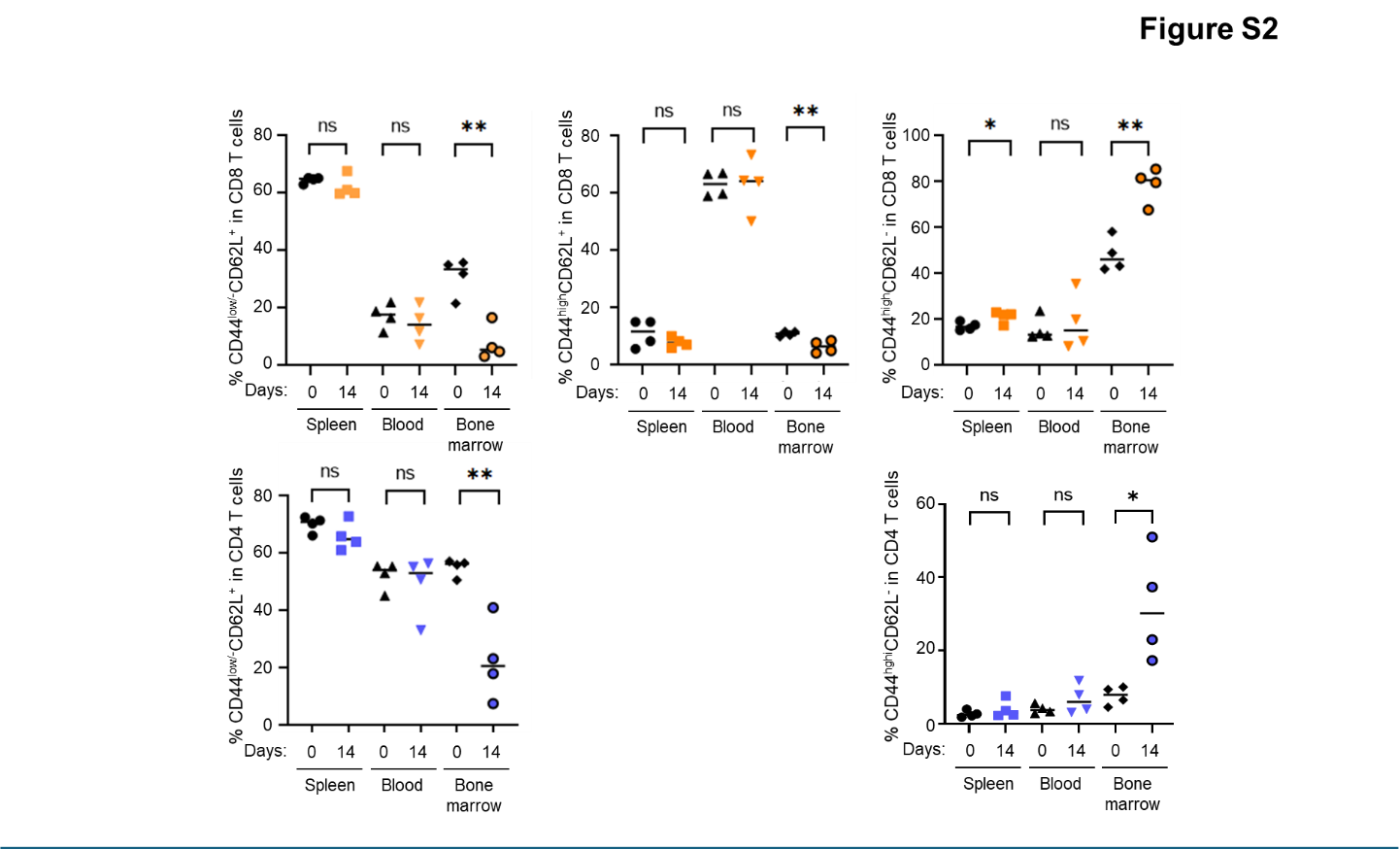


Supplementary Figure 2. Myeloma transplantation altered the frequencies of CD44^high^CD62L^+/-^ and CD44^low/-^CD62L^+^ T cells.

Dot plots show the frequencies of CD44^low/-^CD62L^+^ CD8 T cells, CD44^high^CD62L^+^ CD8 T cells, CD44^high^CD62L^-^ CD8 T cells, CD44^low/-^CD62L^+^ CD4 T cells and CD44^high^CD62L^-^ CD4 T cells in the spleen, peripheral blood and bone marrow in mice on day 14 after myeloma transplantation and non-transplanted mice. ns: not significant, *: p<0.05, **: p<0.01.


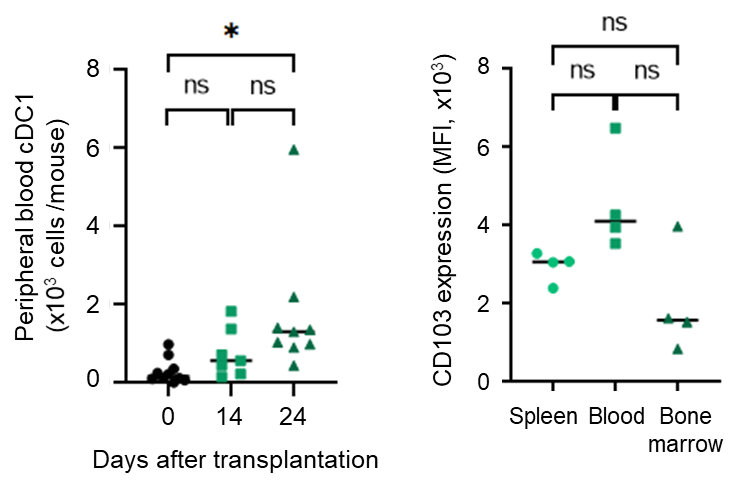


Supplementary Figure 3. Circulating cDC1s increased in the late phase of myeloma expansion.

The numbers of XCR1^+^CD11c^high^ cells in the peripheral blood on days 0, 14 and 24 (A) and the expression of CD103 in cDC1s of the spleen, peripheral blood and bone marrow in mice on day 24 after myeloma transplantation were analyzed by flow cytometry. Data were merged from two independent experiments. N=4-9. ns: not significant, *: p<0.05.
